# Mr. Claudius Galen Wheelhouse

**Published:** 1909-04-24

**Authors:** 


The death is announced, in his eighty-third year, of Mr. Claudius Galen Wheelhouse, F.R.C.S., LL.D., D.Sc. Mr. Wheelhouse was in succession Professor of General Anatomy, Physiology, and Surgery. The high position of the Leeds School of Medicine was to a large extent due to the ability and enthusiasm of Mr. Wheelhouse. On two occasions he held the position of President of the School. In 1864 he was elected Surgeon to the Leeds Infirmary and continued to hold this post for twenty years. In 1885 he was elected direct representative of the profession on the General Medical Council, a position which he held for ten years.

